# Coexistence of Thyrolipomatosis and Tongue Squamous Cell Carcinoma: A Case Report

**DOI:** 10.17925/EE.2023.19.1.103

**Published:** 2023-01-23

**Authors:** Jose Paz-Ibarra, Marcio Concepción-Zavaleta, Daniel Mendoza-Quispe, Jacsel Suárez-Rojas, Katia Rivera Fabián, Diana Deutz-Gómez, Juan Quiroz-Aldave, José Somocurcio Peralta, Tula Ayquipa Arróspide

**Affiliations:** 1. Division of Endocrinology, Hospital Nacional Edgardo Rebagliati Martins, Universidad Nacional Mayor de San Marcos Lima, Peru; 2. Division of Endocrinology, Clínica Javier Prado, Lima, Peru; 3. ADIECS Association for the Development of Student Research in Health Sciences, Universidad Nacional Mayor de San Marcos, Lima, Peru; 4. Universidad Científica del Sur, Lima, Peru; 5. Division of Endocrinology, Hospital Nacional Guillermo Almenara Irigoyen, Lima, Peru; 6. Division of Medicine, Hospital de Apoyo Chepén, Chepén, Peru; 7. Division of Anatomical Pathology, Hospital Nacional Edgardo Rebagliati Martins, Lima, Peru

**Keywords:** Lipomatosis, tongue neoplasms, squamous cell carcinoma, goitre, Peru

## Abstract

Thyrolipomatosis, a diffuse non-neoplastic infiltration of fatty tissue in the thyroid gland, is an extremely rare condition with only about 30 cases reported worldwide. A few of these cases report the concurrency of thyrolipomatosis and malignant neoplasms in the thyroid or colon, but never with tongue cancer. A 44-year-old female patient with an infiltrative tongue mass suggestive of carcinoma presented for an outpatient consultation. Cervical imaging revealed multiple lymphadenopathies and a multinodular goitre with diffuse fatty infiltration, suggestive of thyrolipomatosis. Surgical intervention included partial resection of the tongue and thyroid (left hemiglossectomy and right hemithyroidectomy, respectively) and lymphadenectomy. The thyroid specimen showed diffuse fat metaplasia of the stromal thyroid tissue, confirming incidental thyrolipomatosis. During post-operative follow-up, the patient presented with recurrence of squamous cell carcinoma as indicated by new right-sided thyroid nodules, left-sided lymphadenopathies with confirmatory biopsy, and a growing neck mass that became infected. The patient developed septic shock and later died. Thyrolipomatosis causes thyroid swelling and can be clinically detected as goitres or as an incidental finding. Diagnosis is suggested by cervical imaging (ultrasonography, computed tomography or magnetic resonance), but confirmation is histological after thyroidectomy. Although thyrolipomatosis is benign, it could develop concurrently with neoplastic diseases, especially on embryologically related tissues (e.g. thyroid and tongue). This case report is the first in the literature describing the coexistence between thyrolipomatosis and tongue cancer in an adult Peruvian patient.

**Article highlights**

- There have only been about 30 cases of thyrolipomatosis reported worldwide
- Its underlying pathophysiology or its association with certain cancers is unknown
- Few cases reported its concurrency with malignant neoplasms, such as tongue cancer
- Diagnosis is confirmed by anatomopathological study after thyroidectomy

Thyrolipomatosis is a rare condition defined as a diffuse non-neoplastic infiltration of fatty tissue in the thyroid gland.^1^ Although fatty infiltration is common in other glands (e.g. salivary glands, parathyroids, thymus and pancreas), it is rare in the thyroid gland.^2^ If present, it is most frequently nodular (i.e. thyrolipoma) rather than diffuse (i.e. thyrolipomatosis).^3^ Thus, since the first case of thyrolipomatosis was reported in 1942 by Dhayagude,^4^ only about 30 cases have been published worldwide.^5–7^ In addition, few of these cases showed concurrency of thyrolipomatosis and malignant neoplasms in the thyroid or colon, but never with tongue cancer. In this report, we present the case of an adult Peruvian patient with squamous cell carcinoma of the tongue and incidental thyrolipomatosis, along with a literature review.

## Case report

A 44-year-old female patient was assessed at the head and neck surgery outpatient department at a tertiary care public hospital in Lima, Peru, in November 2021. Her main complaint was a tongue mass that had progressively enlarged over the past 1.5 years, which was associated with dysphagia to solids and weight loss. Her history included non-toxic multinodular goitre and juvenile rheumatoid arthritis that were diagnosed when she was in her twenties and treated with leflunomide. There was no history of radiation exposure or any contributing family history. Physical examination revealed a left-sided pearly mass on the tongue (3 cm × 1.5 cm) suggestive of carcinoma, goitre grade IB (i.e. palpable goitre with a painless, mobile, right-sided thyroid nodule), palpable bilateral cervical lymphadenopathies, cervical skin without changes, undernutrition (body mass index 17.8 kg/m^2^) and vital signs in normal range. Laboratory examinations revealed mild microcytic anaemia (haemoglobin: 11.4 mg/ dL; mean corpuscular volume: 73 fL). Cervical ultrasonography revealed a predominantly left-sided multinodular goitre, a left-sided thyroid nodule (31 mm × 22 mm; mixed solid and cystic content; slightly hypoechogenic) and multiple bilateral lymphadenopathies (i.e. a right-side node of 24 mm × 5.9 mm, another right-side node of 12 mm × 4 mm, and a left-sided node of 27 mm × 11 mm). Fine-needle aspiration (FNA) of the lymph nodes revealed squamous cell carcinoma in the left adenopathy and reactive lymphoid hyperplasia in the right.

**Figure 1:**
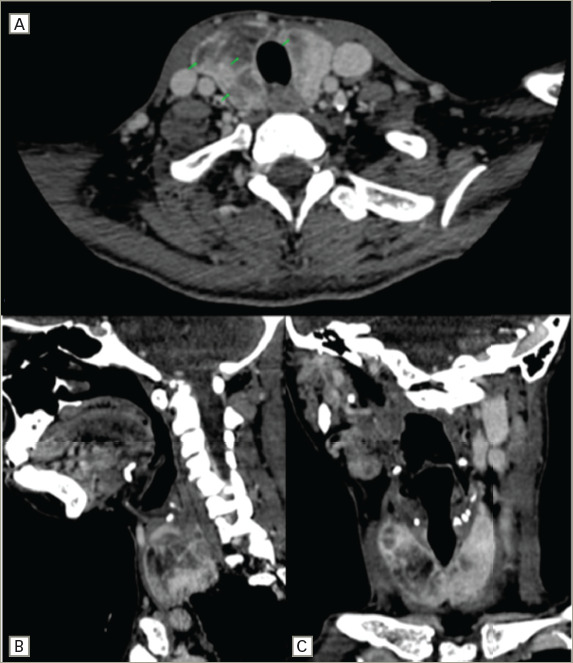
Pre-operative cervical computed tomography scan with contrast

*A: Axial plane showing diffuse hypodense areas on both thyroid lobes, predominantly right sided. B and C: Coronal planes showing diffuse thyromegaly with multiple septa, cystic content, and bilateral fatty acid infiltration.*

**Figure 2:**
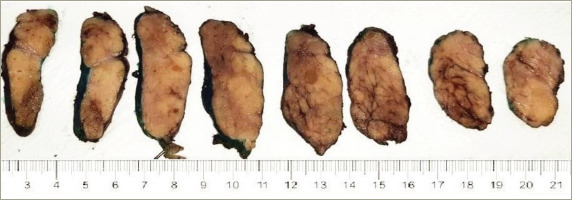
Serial sections of the right thyroid lobule specimen

*The internal surface exhibits yellowish colour and is slightly nodular.*

**Figure 3:**
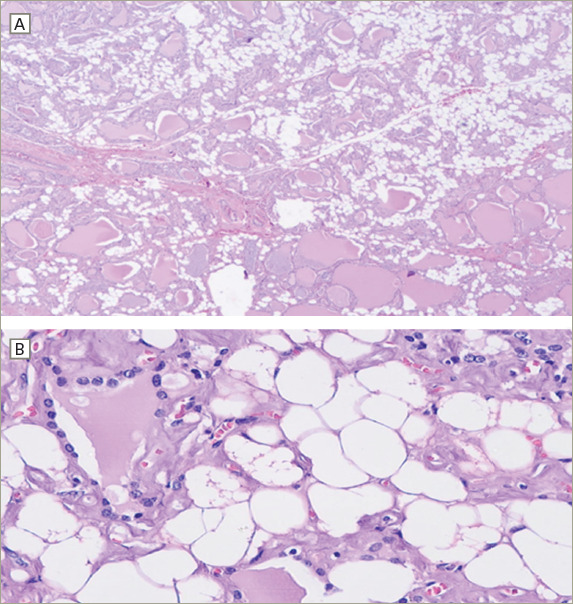
Haematoxylin and eosin-stained microphotographs of the resected right thyroid lobule

*A: Panoramic view (10×) showing few thyroid follicles among a marked proportion of fatty tissue. B: At higher magnification (40×), mature adipocytes and spared thyroid follicles are observed.*

**Figure 4:**
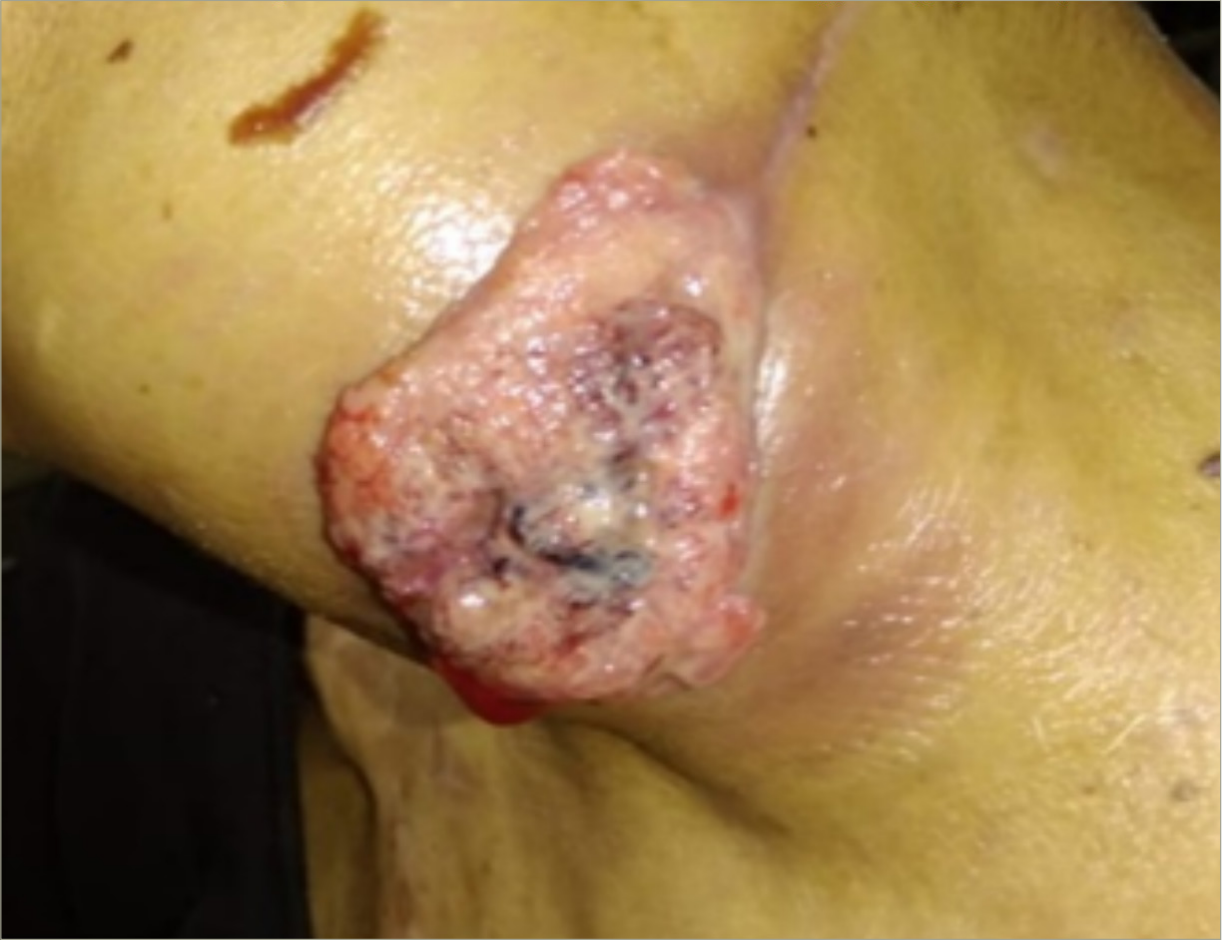
Left neck mass during post-operative follow-up

*The mass has an infiltrative bloody aspect with central necrosis.*

The cervical computed tomography (CT) scan with oral contrast staged the squamous cell carcinoma of the tongue as cT3 N2b Mx G1 according to TNM classification (*Figure 1*). The CT scan also revealed infiltration of the parotids, lymph node conglomerate in the left cervical chain with an infiltrative appearance, and diffuse thyromegaly with multiple septa, cystic content and bilateral fatty infiltration.

The patient was admitted for surgical intervention that consisted of partial resection of the tongue and thyroid (i.e. left hemiglossectomy and right hemithyroidectomy, respectively) alongside a radical modified cervical lymphadenectomy (type I on the left, type III on the right), and a procedure for mandibular correction (i.e. symphyseal osteotomy). Gross examination of the thyroid specimen (*Figure 2*) showed a reddish, right thyroid lobule, normal weighted (15 g), increased in size (6 cm × 3.5 cm × 2 cm) of elastic consistency containing colloid cysts and remaining homogeneous parenchyma. Histologic examination of the thyroid specimen (*Figure 3*) showed non-neoplastic macrofollicular colloidal hyperplasia with cystic degeneration and diffuse fat metaplasia of stromal thyroid tissue, confirmatory of thyrolipomatosis. Biopsy was Congo red negative, excluding amyloid infiltration.

**Table 1:** Epidemiological features of reported cases of thyrolipomatosis

Reference	Sex	Age, y	Comorbidity	Symptoms	Signs	Ultrasound	Thyroid scintigraphy	Thyroid surgery	Histology
Arslan (1999)^8^	Male	38	NS	Cervical mass	Diffuse goitre	NR	Bilateral diffuse enlargement and mildly decreasing radiotracer in left lobe	Subtotal thyroidectomy	Mature adipose infiltrating tissue
Di Scioscio (2008)’	Male	68	Chronic kidney failure, psoriasis	Cervical mass, dyspnoea	NS	Diffuse goitre, heterogeneous echogenicity	Enlargement of thyroid, with irregularly reduced uptake	NR	NR
Ge (2009)^3^	Female	67	Morbid obesity, diabetes, CKD	Cervical mass, dysphagia	Asymmetrical goitre	NR	2 cm hypofunctioning nodule in left thyroid	Left hemithyroidectomy	Mature adipose diffuse-infiltrating tissue
Ge (2009)^3^	Female	59	Hypothyroidism, thyroid nodules	NS	NS	Heterogeneous echogenicity in left thyroid	NR	Total thyroidectomy	Mature adipose infiltrating tissue, papillary carcinoma
Gonulalan (2012)™	Male	43	Amyloidosis, CKD	Cervical mass, dyspnoea	Asymmetrical goitre	Heterogeneous echogenicity	Diffuse uptake of radioactivity and a cold area in the superior part of the left lobe	Total thyroidectomy	Mature adipose infiltrating tissue
Cacchi (2013)^11^	Male	51	Multinodular goitre	NS	NS	NR	NR	Total thyroidectomy	Mature adipose infiltrating tissue, papillary carcinoma
Lo and Donaldson (2013)™	Female	52	NS	Cervical mass, dyspnoea	NS	Diffuse goitre, heterogeneous echogenicity	NR	NR	NR
Jacques and Stearns (2013)™	Male	55	Crohn's disease, amyloidosis, CKD	2-year cervical mass	Diffuse goitre	Diffuse goitre, hyperechogenicity	NR	Total thyroidectomy	Mature adipose infiltrating tissue, amyloid goitre
Sanuvada (2014)™	Female	32	Toxic multinodular goitre	2-year midline cervical mass	Asymmetrical goitre	Diffuse goitre, heterogeneous echogenicity, nodules in right lobe	Hyperfunctioning enlarged thyroid gland with grossly enlarged right lobe	Total thyroidectomy	Mature adipose infiltrating tissue
Kumar (2016)™	Male	73	Multinodular goitre	Cervical mass	Diffuse goitre	Diffuse goitre and nodules	NR	Total thyroidectomy	Mature adipose infiltrating tissue
Bell (2016)^7^	Female	36	Epilepsy, rheumatoid arthritis, bone marrow aplasia, CKD	Cervical mass, dyspnoea	Nodular goitre	Thyroid nodules	NR	Total thyroidectomy	Mature adipose infiltrating tissue, amyloid goitre
Loh (2017)™	Male	67	CKD	Dysphonia, cervical mass	NS	Diffuse goitre, nodule in right lobe	NR	First right and then left hemithyroidectomy	Mature adipose infiltrating tissue, follicular carcinoma
Stanawayand Lam (2019)^17^	Male	19	Thymolipoma	Cervical mass, dysphagia	Asymmetrical goitre	Left lobe enlargement	NR	Left hemithyroidectomy	Mature adipose infiltrating tissue
Ishida (2017)™	Female	68	Diabetes mellitus and angina pectoris	Cervical mass	NS	NR	NR	Total thyroidectomy	Mature adipose infiltrating tissue
Balasubramanian, (2018)™	Female	49	Graves' disease	Cervical mass	Diffuse goitre	NR	Hyperfunctioning enlarged thyroid gland	Total thyroidectomy	Mature adipose infiltrating tissue
Lopez-Munoz (2019)^20^	Female	48	Rheumatoid arthritis, amyloidosis, CKD, subclinical hyperthyroidism	Cervical mass, dysphagia	NS	Diffuse goitre	NR	Total thyroidectomy	Mature adipose infiltrating tissue, amyloid perivascular infiltration
Campion (2021)^21^	Male	40	Myasthenia gravis, thymoma	Cervical mass, dysphagia	Mass in left lobe of thyroid	Thyroid nodules	NR	Total thyroidectomy	Mature adipose infiltrating tissue
Rodrigues (2021)^3^	Male	46	Hypogonadotropic hypogonadism	Dysphagia	Diffuse goitre	Diffuse goitre, thyroid nodules	NR	Total thyroidectomy	Mature adipose infiltrating tissue, amyloid perivascular infiltration

*CKD = chronic kidney disease; CT = computed tomography; NR = not reported; NS = not specified.*

During post-operative follow-up, the patient was euthyroid (thyroidstimulating hormone 0.733 mU/L). Three months post-operatively, a control cervical ultrasonography suggested metastasis, after images showed a left-sided thyroid nodule with an American College of Radiology Thyroid Imaging Reporting and Data System score of 4 (18 mm × 13 mm), a left-sided lymphadenopathy (10 mm × 5 mm) and a left cervical mass (43 mm × 26 mm); all of these pathologies had malignant features (e.g. ill-defined borders, heterogenous content and marked Doppler vascularity). Recurrent cancer was confirmed by a FNA cytology of the lymphadenopathy that showed squamous malignant cells. The left neck mass grew rapidly and became painful and bloody (*Figure 4*). However, as the referred mass was highly vascularized and was located around major vessels (e.g. left carotid artery, subclavian artery and jugular vein, according to angiotomography), the patient was a poor surgical candidate due to high risk of massive bleeding. The multidisciplinary medical team suggested palliative locoregional radiotherapy to reduce mass size and gastrostomy due to dysphagia. Five months post-operatively, the left neck mass became infected; the patient was admitted to hospital and received broad-spectrum antibiotics (meropenem and vancomycin), but septic shock progressed and the patient died.

## Discussion

This case report is the first in literature documenting concurrent thyrolipomatosis and squamous cell carcinoma of the tongue in an adult Peruvian patient. This presentation suggests that, although thyrolipomatosis is a benign and incidental condition, it may be concurrent with malignant neoplasms.

*Table 1* summarizes reported cases and lists the epidemiological features of thyrolipomatosis. For example, there is no predominance by sex (56% male), its occurrence is mostly in middle-aged adults (mean age 51 years; range 44–57 years) and it is usually associated with systemic conditions like chronic kidney failure (39%) and amyloidosis (17%).^3,6–21^

Thyrolipomatosis is a benign condition because the fatty infiltration of the thyroid gland is diffuse but non-neoplastic. However, some case reports suggest the coexistence of thyrolipomatosis with malignant neoplasms such as papillary thyroid carcinoma or colon cancer.^6,22,23^ As mentioned, this report is the first published case of a patient with thyrolipomatosis with concurrent squamous cell carcinoma of the tongue. *Table 1* confirms that it may coexist with malignancies in less than 20% of cases.

A hypothesis of causality between thyrolipomatosis and tongue cancer is not reasonable, since these entities have different histopathological findings; nonetheless, the organs in which both conditions are located (i.e. the thyroid and tongue) are embryologically related and may partly explain their coexistence. During the pre-natal period, the thyroid gland arises near the base of the tongue and migrates caudally; it remains attached to the tongue via the thyroglossal duct, which almost always obliterates entirely.^24^

The underlying pathophysiology of thyrolipomatosis is unclear; it may involve either embryological growth of fatty tissue in the thyroid gland,^25^ fatty metaplasia of stromal fibroblasts in response to hypoxia^14^ or a mutation of a mitochondrial protein.^25^ As thyrolipomatosis causes thyroid swelling, patients usually present with a progressive goitre, either diffuse or nodular, which may be asymptomatic or cause compressive symptoms such as dyspnoea, dysphagia or dysphonia.^2,26^ Our patient presented with dysphagia due to another condition (a lymphadenopathy). Most patients, as with our case, have normal thyroid function (see *Table 1*), although some may present with elevated^14,27^ or decreased 3 thyroid hormone levels.

On work-up, cervical imaging (ultrasonography, CT or magnetic resonance) can suggest adipose thyroid tissue infiltration and is recommended in the pre-operative diagnosis. A CT scan will show an enlarged thyroid gland with areas of fatty attenuation due to lipomatous infiltration intermixed with areas of greater attenuation corresponding to thyroid parenchyma.^16,19^ Pre-operatively, FNA cytology could also show adipocytes in the thyroid tissue.^14,19,28^ A definitive diagnosis of thyrolipomatosis is established by histological examination of surgical thyroid specimens after thyroidectomy. Diffuse infiltration of mature adipocytes between sparse thyroid follicles is characteristic,^10^ as shown in this case.

The differential diagnosis should include other thyroid lesions, both neoplastic and non-neoplastic, that contain mature fatty tissue. Examples of neoplastic lesions include thyrolipoma, papillary carcinoma and follicular carcinoma, while examples of non-neoplastic lesions include an adenomatous nodule, thyrolipomatosis, amyloid goitre, dyshormonogenetic goitre and Hashimoto’s thyroiditis.^18^

Thyrolipoma is the most common fat-containing lesion of the thyroid gland. The characteristic histopathological feature is the presence of mature fatty tissue in the follicular adenoma and a fibrous capsule around the tumour.^18^ Amyloid goitres are also a relatively common nonneoplastic thyroid lesion that contains fat. The presence of amyloid among non-neoplastic thyroid follicles is characteristic of this condition, and Congo red staining is positive.^20,29^

## Conclusion

Diffuse infiltration of fatty tissue in the thyroid gland is an exceedingly rare, benign and often incidental condition. In the work-up of thyrolipomatosis, concurrent systemic or neoplastic diseases such as tongue cancer could be investigated.
